# Age at Menarche in Women With Bipolar Disorder: Correlation With Clinical Features and Peripartum Episodes

**DOI:** 10.3389/fpsyt.2020.00851

**Published:** 2020-09-04

**Authors:** Gianluca Rosso, Elena Aragno, Emina Mehanović, Gabriele Di Salvo, Giuseppe Maina

**Affiliations:** ^1^ Department of Neuroscience “Rita Levi Montalcini,” University of Torino, Torino, Italy; ^2^ Psychiatric Unit, San Luigi Gonzaga University Hospital, Torino, Italy

**Keywords:** bipolar disorder, women, reproductive cycles, menarche, peripartum episodes

## Abstract

**Background:**

Bipolar disorder (BD) is related to reproductive cycles. However, findings on putative correlation between age at menarche and course of BD are particularly scarce and conflicting. The aim of the present study is to analyze the relationship between age at menarche and characteristics of BD, including comorbid medical conditions and peripartum mood episodes.

**Methods:**

The study sample consists of 288 women with BD type I, II, or not otherwise specified (NOS). Age at menarche was both considered as a continuous variable and categorized into three groups: early menarche (≤11 years), normal menarche (12–14 years), and late menarche (≥15 years). The study focused on two sets of comparisons, by age at menarche and women with vs. without children. Spearman correlation matrix was produced to calculate correlations between the variables of interest. Socio-demographic and clinical characteristics between early, normal and late menarche, and women with vs. without children were examined through descriptive statistics. Finally, adjusted logistic regression analysis was run to examine the association between variables.

**Results:**

Out of 288 women included in the study, 21.5% had early menarche, 55.6% had normal, and 22.9% had late menarche. Women with early menarche had higher rates of metabolic syndrome compared to women with normal menarche even after adjustment for age. The subgroup of women with children does not present clinical differences compared to women without children except a lower rate of psychiatric comorbidities. At least one mood episode with peripartum onset occurred in 29.6% of the women with children. After controlling for confounding variables, women with late menarche were associated with lower probability of BD peripartum episodes compared to women with normal menarche.

**Conclusion:**

Age at menarche may be related to specific characteristics of women with BD. The results deserve to be deepened in further studies.

## Introduction

Bipolar disorder (BD) is related to reproductive cycles. Women with BD may experience mood symptoms during premenstrual, peripartum, and perimenopausal periods. Above all, there is strong, clear and consistent evidence of a specific correlation between childbirth and acute episodes of BD (1). Di Florio and colleagues reported, respectively, a 50% risk of a perinatal major affective episode per pregnancy/postpartum period in women with bipolar I disorder and a 40% risk in women with bipolar II disorder (2). The risk of BD recurrences in the postpartum period is further increased by treatment discontinuation during pregnancy: Viguera and colleagues found that postpartum recurrences in pregnant women with BD after lithium discontinuation were 2.9 times more frequent than recurrences in nonpregnant women (3); moreover, we found that the 75% of a large sample of BD women who were medication-free during their pregnancies experienced mood disorders after delivery (4).

While the peripartum period is clearly recognized as a high-risk period for recurrences in patients with BD, the impact of menstrual cycle and menopause on the course of illness is up for debate (5–7). A cross-sectional study provided preliminary evidences that Premestrual dysphoric disorder is frequently comorbid with bipolar spectrum disorders, notably type II BD and cyclothymia (8, 9). More recently, Teatero and colleagues suggested that a subgroup of women with BD, possibly those with hormonal sensitivity, experience menstrual cycle effects on depressive, hypomanic, and manic episodes. These phase-episode effects appear to be heterogeneous and may have implications for treatment (10).

With regard to the impact of menopause on BD, results are conflicting. It has been shown that menopause may improve, worsen, or not affect the course of illness (11). Interestingly, it seems that emotional disturbances during the menopausal transition in women with BD could lead to a rapid cycling course of the disorder (12). Moreover, data suggest that women with BD who are not using hormone replacement therapy are more likely to report worsening of mood symptoms during perimenopause (13).

Findings on putative correlation between age at menarche and course of BD are particularly scarce and conflicting. Freeman and colleagues (14) found that, in many patients, the onset of BD can occur both before menarche and within 1 year of menarche. Another study showed a link between age at menarche and mood disorders suggesting that earlier menarche predicts more depression in adolescence (15). Conversely, in a recent investigation focused on relationship between timing of menarche, pubertal mental health, and psychiatric disorders, the age at menarche was significantly higher in bipolar group than schizophrenic, depressed, and control groups. The authors concluded that pubertal timing and psychological problems during adolescence might be related to the age at onset of psychiatric disorders, and the association was especially strong for BD (16).

Whether age at menarche has long-term effects on the course of mental illnesses is even more obscure. A study on girls at age 24, followed up to 10 years, found that those reporting early pubertal timing had greater risk of lifetime psychiatric disorders compared to the on-time and late developers (17). Conversely, in a recent 3-year follow-up study among adolescent girls, the relation between age at menarche and mental distress disappeared among 15- to 18-year-old girls (18). To our knowledge, today, there are no studies exploring the relationship between age at menarche and long-term course of BD. Furthermore, research is lacking on the correlation between menarche time and medical diseases that commonly afflict patients with BD such as metabolic syndrome whose prevalence is higher than the general population. (19, 20). Lastly, it is not known whether the risk of peripartum mood episodes in women with BD may be influenced by the age at menarche. Therefore, the aim of the present study is to analyze the relationship between age at menarche and characteristics of BD, including comorbid medical conditions and peripartum mood episodes.

## Methods

### Sample

This is a cross-sectional observational study involving in- and out-patients with diagnosis of BD consecutively referred to the Psychiatric Unit of San Luigi Gonzaga University Hospital of Orbassano (Turin, Italy), from January 2014 to June 2018. For the purpose of the present study, only female subjects were considered.

Two hundred ninety-five women with BD were asked to participate; five refused their consent. Among the 290 subjects recruited, two (0.7%) were excluded from the research due to lack of data on age at menarche. The analysis was performed on 288 women with BD I, II, or not otherwise specified (NOS) according to the criteria of Diagnostic and Statistical Manual of Mental Disorders–Fifth Edition (DSM-5) (21).

The study design was reviewed by the local ethics committee. Written informed consent was obtained from participants after the procedure had been fully explained.

Certified psychiatrists or residents in psychiatry supervised by senior psychiatrists performed the clinical assessment of subjects. Socio-demographic data (age, marital status, years of education, and occupational status), clinical features of BD (age at onset and other course specifiers such as type of bipolar cycle), and comorbid psychiatric disorder were obtained through the administration of a semi-structured interview that we developed and used in regular clinical practice and in previous studies as well (22). In addition, information on smoking and alcohol, medical conditions (including metabolic syndrome), and reproductive cycle event data were collected through individual patient medical records. The metabolic syndrome was diagnosed in the patients on the basis of the concurrent presence of at least three of the following conditions: abdominal obesity, hypertriglyceridemia, low HDL-cholesterol, high blood pressure, and high fasting glucose (23).

### Statistical Analysis

The study focused on two sets of comparisons, by age at menarche and women with vs. without children (n = 288). Age at menarche was both considered as a continuous variable and categorized into three groups, defined according to previous studies (24): early menarche (EM) (≤11 years), normal menarche (NM) (12–14 years), and late menarche (LM) (≥15 years). Spearman correlation matrix was produced to calculate correlations between variables of interest. Socio-demographic and clinical characteristics between EM, NM, and LM, and women with vs. without children were examined through descriptive statistics. Median (range) and frequencies (percentages) were used to summarize the data on subject characteristics. Pearson chi-square and Fisher Exact tests were used to examine the association between categorical variables. Due to non-parametric nature of the data, Kruskal-Wallis and Mann-Whitney were applied to test the association between studied correlates. Bonferroni correction for Kruskal-Wallis post-hoc multiple comparison testing was performed. Further, the associations of age at menarche with metabolic syndrome (controlling for age) and BD episodes in the peripartum period (controlling for age, number of children, age at onset of BD, type of BD and total number of affective episodes), as well as the relationship between clinical characteristics and women with children (controlling for age, age at onset of BD, and duration of untreated illness) were examined through adjusted logistic regression models. The potential confounding factors included in the adjusted logistic regression models were selected according to previous studies and clinical practice. The regression model on woman with children was run for each clinical characteristic separately. In case of collinearity (r ≥ 0.6), only one covariate was chosen for the inclusion in the model. Missing data was <2.4% for tested variables. Adjusted odds ratios (AOR), 95% confidence intervals, and p-value of <.05 were estimated as the measures of association. All statistical analyses were carried out using IBM SPSS software version 25 (25).

## Results

### Age at Menarche

Results from Spearman correlations matrix are reported in **Table 1**. Significant positive correlations (p <.05) emerged between age at menarche and number of lifetime depressive episodes (r = 0.118), duration of untreated illness (r = 0.155) and age at first treatment for BD (r = 0.164) (**Table 1**). Out of 288 women with BD included in the study, 21.5% had EM, 55.6% had NM, and 22.9% had LM. The median age of study participants and the median age at menarche were, respectively, 51 (16–81) years and 13 (9–17) years (mean age at menarche 12.6 ± 1.3 years). About 58% of women had BD type II, the median age at the onset and the median duration of BD were 27 (12–72) and 20 (0.1–57) years, respectively. Adjusting for Bonferroni correction, women with NM were younger than those with LM [47 (17–81) vs. 55 (32–75), p = .013], whereas women with EM had higher levels of total cholesterol compared to those with NM [217 (125–377) vs. 193 (108–341), p = .024]. Moreover, women with EM had higher proportion of metabolic syndrome compared to normal and LM subgroups (41.7 vs. 24.2 vs. 28.1%, respectively, p = .039) (**Table 2**). After adjustment for age, EM was associated with 2 times higher odds of metabolic syndrome compared to NM group (OR, 2.07; 95% CI, 1.07–3.99), whereas LM was not statistically significant (**Table 3**).

**Table 1:** Spearman correlation matrix.

	Age at menarche	Lifetime manic episodes (number)	Lifetime hypomanic episodes (number)	Lifetime major depressive episodes (number)	Lifetime mixed episodes (number)	Lifetime total affective episodes (number)	Age at first major depressive episode	Age at BD onset	Duration of untreated illness	Age at first treatment for BD
Lifetime manic episodes (number)	-0.098										
Lifetime hypomanic episodes (number)	0.087	-.514*									
Lifetime major depressive episodes (number)	.118*	-0.091	.496*								
Lifetime mixed episodes (number)	-0.036	.318*	-.324*	-0.083							
Lifetime total affective episodes (number)	0.073	0.079	.581*	.909*	0.043						
Age at first major depressive episode	0.035	-0.106	-0.07	-.233*	-0.067	-.249*					
Age at BD onset	0.054	-.126*	-0.038	-.157*	-0.071	-.186*	.969*				
Duration of untreated illness	.155*	-.220*	.291*	.397*	-.169*	.323*	-.267*	-.268*			
Age at first treatment for BD	.164*	-.318*	.189*	.150*	-.173*	0.063	.520*	.533*	.588*		
Total cholesterol	0.022	-0.112	0.087	0.091	-0.117	0.064	0.101	0.094	.141*	.165*	

*Correlations with p-value < 0.05.

**Table 2:** Socio-demographic and clinical characteristics by age at menarche.

Characteristics	Early menarche (n = 62, 21.5%)	Normal menarche (n = 160, 55.6%)	Late menarche(n = 66, 22.9%)	Overall(n = 288)	X^2/^H test	P-value
Age (year), median (range)	55 (16–80)	47 (17–81)	55 (32–75)	51 (16–81)	8.568	**0.013^c^**
Type of BD, n (%)						
BD I	26 (41.9)	65 (40.6)	22 (33.3)	113 (39.2)		
BD II	35 (56.5)	91 (56.9)	42 (63.6)	168 (58.3)		
BD NOS	1 (1.6)	4 (2.5)	2 (3.0)	7 (2.4)	1.589	0.832^b^
Age at onset (year), median (range)	30 (12–60)	25 (14–72)	29 (14–69)	27 (12–72)	5.595	0.061**^c^**
Duration of illness (year), median (range)	21 (0.1–52)	17.5 (0.1–57)	23.5 (0.1–52)	20 (0.1–57)	4.662	0.097**^c^**
Duration of untreated illness (year), median (range)	8 (0.1–49)	11 (0.1–51)	14 (0.1–43)	12 (0.1–51)	5.394	0.067**^c^**
Lifetime total affective episodes, median (range)	7 (1–45)	8.5 (1–40)	8 (1–66)	8 (1–66)	4.683	0.096**^c^**
Lifetime major depressive episodes, median (range)	4 (0–25)	5 (0–30)	5 (0–33)	4 (0–33)	6.016	0.072
Lifetime hypomanic episodes, median (range)	1.5 (0–20)	2 (0–15)	2 (0–33)	2 (0–33)	1.014	0.602**^c^**
Lifetime psychiatric comorbidities, n (%)						
No	41 (66.1)	91 (56.9)	43 (65.2)	175 (60.8)		
Yes	21 (33.9)	69 (43.1)	23 (34.8)	113 (39.2)	2.296	0.317^a^
Lifetime medical comorbidities, n (%)						
No	17 (27.4)	61 (38.1)	17 (25.8)	95 (33.0)		
Yes	45 (72.6)	99 (61.9)	49 (74.2)	193 (67.0)	4.341	0.114^a^
Family history of BD, n (%)						
No	43 (69.4)	123 (77.4)	50 (75.8)	216 (75.3)		
Yes	19 (30.6)	36 (22.6)	16 (24.2)	71 (24.7)	1.546	0.462^a^
Metabolic syndrome, n (%)						
No	35 (58.3)	119 (75.8)	46 (71.9)	200 (71.2)		
Yes	25 (41.7)	38 (24.2)	18 (28.1)	81 (28.8)	6.472	**0.039** ^a^
Total cholesterol (mg/dl), median (range)	217 (125–377)	193 (108–341)	207 (113–374)	199 (108–377)	8.292	**0.024^c^**

^a^Pearson chi-square test.

^b^Fisher Exact test performed.

^c^Kruskal-Wallis test for non-parametric data (adjusted for Bonferroni correction). Bold font indicates statistical significance.

**Table 3:** Age at menarche and metabolic syndrome.

	COR^a^(95% CI)	P-value	AOR^b,c^(95% CI)	P-value
Age of menarche				
Normal	1		1	
Early	2.24 (1.19–4.20)	0.012	2.07 (1.07–3.99)	0.030
Late	1.23 (0.64–2.36)	0.544	1.01 (0.51–2.00)	0.969

^a^COR, crude odds ratios.

^b^AOR, adjusted odds ratios (adjusted for age).

^c^The analytical sample included 281 subjects.

#### Woman With and Without Children

**Figure 1:**
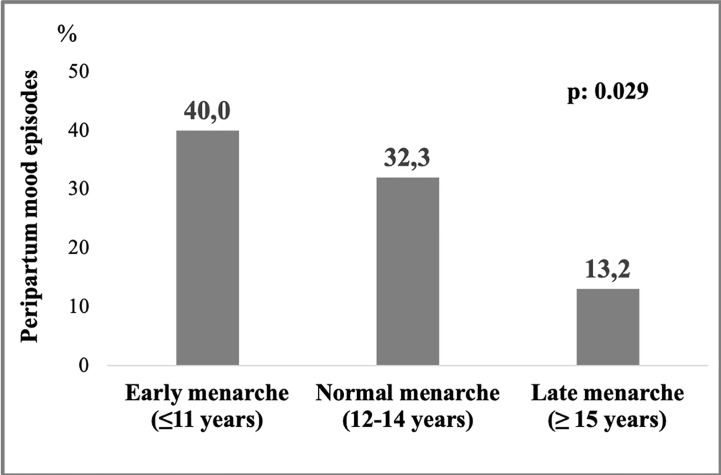
Differences in peripartum mood episodes between early, normal and late menarche subgroups.

Out of 288 women included in the study, 58.7% had children (**Table 4**). Women with children had significantly higher proportion of BD type II (65.7 vs. 47.9%, p = .008), lifetime medical comorbidities (74.6 vs. 56.3%, p = .001), and metabolic syndrome (37.0 vs. 17.2%, p = <.001), but lower lifetime psychiatric comorbidities (30.8 vs. 51.3%, p<.001) compared to women without children. Moreover, they were older [57 (27–81) vs. 45 (16–81), p<.001], they had a later onset of BD [30 (14–72) vs. 25 (12–68), p = .001] and a higher number of depressive [5 (0–33) vs. 4 (0–20), p = .011] and hypomanic episodes [2 (0–33) vs. 1.5 (0–15), p = .014]. The duration of illness [23 (0.1–57) vs. 15 (1–52), p<.001] and untreated illness [16 (0.1–51) vs. 7 (0.1–44), p<.001] was also significantly higher for women with children compared to woman without children. After controlling for confounding factors, only lifetime psychiatric comorbidity maintained its significance (OR: 0.56, 95% CI: 0.32–0.98) (**Table 5**). Out of 169 women with children, 29.6% had at least one BD peripartum episode. The highest proportion of peripartum episodes was observed in women with EM compared to women with NM and LM (40.0 vs. 32.3 vs. 13.2%, p = .029) (**Figure 1**). After adjustment, the LM was associated with lower probability of BD episodes during the peripartum period compared to NM (OR: 0.33, 95% CI: 0.11–0.99) (**Table 6**).

**Table 4:** Differences in socio-demographic and clinical characteristics between women with and without children.

Characteristics	Women with children(n = 169, 58.7%)	Women without children(n = 119, 41.3%)	X^2/^U test	P-value
Age (year), median (range)	57 (27–81)	45 (16–81)	5346.5	**<0.001^c^**
Type of BD, n (%)				
BD I	55 (32.5)	58 (48.7)		
BD II	111 (65.7)	57 (47.9)		
BD NOS	3 (1.8)	4 (3.4)	9.208	**0.008^b^**
Age at onset of BD (year), median (range)	30 (14–72)	25 (12–68)	7719.0	**0.001^c^**
Duration of illness (year), median (range)	23 (0.1–57)	15 (1–52)	7239.0	**<0.001^c^**
Duration of untreated illness (year), median (range)	16 (0.1–51)	7 (0.1–44)	6609.0	**<0.001^c^**
Lifetime total affective episodes, median (range)	8 (1–66)	7 (1–35)	9039.0	0.143^c^
Lifetime major depressive episodes, median (range)	5 (0–33)	4 (0–20)	8299.5	**0.011** ^c^
Lifetime hypomanic episodes, median (range)	2 (0–33)	1.5 (0–15)	8362.5	**0.014** ^c^
Lifetime psychiatric comorbidities, n (%)				
No	117 (69.2)	58 (48.7)		
Yes	52 (30.8)	61 (51.3)	12.298	**<0.001** ^a^
Lifetime medical comorbidities, n (%)				
No	43 (25.4)	52 (43.7)		
Yes	126 (74.6)	67 (56.3)	10.526	**0.001** ^a^
Family history of BD, n (%)				
Metabolic syndrome, n (%)				
No	104 (63.0)	96 (82.8)		
Yes	61 (37.0)	20 (17.2)	12.922	**<0.001** ^a^
Total Cholesterol (mg/dl), median (range)	209 (113–377)	188 (108–281)	5659.5	0.063

^a^Pearson chi-square test.

^b^Fisher Exact test performed.

^c^Mann-Whitney U test for non-parametric data. Bold font indicates statistical significance.

**Table 5:** Clinical correlates of women with children.*

Characteristics	COR (95% CI)^a^	AOR (95% CI)^b,c^
Type of BD		
BD I	1	1
BD II	2.05 (1.26–3.35)	1.59 (0.92–2.75)
BD NOS	0.79 (0.17–3.70)	1.06 (0.19–5.96)
Lifetime psychiatric comorbidities		
No	1	1
Yes	0.42 (0.26–0.69)	**0.56 (0.32**–**0.98)**
Lifetime medical comorbidities		
No	1	1
Yes	2.27 (1.38–3.75)	1.25 (0.70–2.22)
Metabolic syndrome		
No	1	1
Yes	2.82 (1.58–5.01)	1.63 (0.86–3.11)
Lifetime major depressive episodes (number)	1.07 (1.01–1.14)	1.04 (0.97–1.11)
Lifetime hypomanic episodes (number)	1.10 (1.02–1.19)	1.07 (0.98–1.17)

*The regression models were run separately for each of the clinical characteristics

^a^COR, crude odds ratios.

^b^AOR, adjusted odds ratios (adjusted for age, age at onset of BD, and duration of untreated illness).

^c^The analytical samples consist of 281 observations for all characteristics, except metabolic syndrome (n = 274). Bold font indicates statistical significance.

**Table 6:** Age at menarche by BD episodes in peripartum.

	BD episodes in peripartum			COR^a^(95% CI)	P-value	AOR^b,c^(95% CI)	P-value
	Yes(n = 50)	No(n = 119)	Overall(n = 169)				
Age at menarche, n (%)							
Normal	31 (62.0)	65 (54.6)	96 (56.8)	1		1	
Early	14 (28.0)	21 (17.6)	35 (20.7)	1.40 (0.63–3.11)	0.412	2.00 (0.79–5.07)	0.143
Late	5 (10.0)	33 (27.7)	38 (22.5)	0.32 (0.11–0.89)	0.030	0.33 (0.11–0.99)	0.049

^a^COR, crude odds ratios.

^b^AOR, adjusted odds ratios (adjusted for age, number of children, age at onset of BD, type of BD, lifetime total number of episodes).

^c^The analytical sample included 169 observations.

## Discussion

To our knowledge, this is the first study focusing on the association between age at menarche and long-term course of illness in women with BD. Given the known relationship between reproductive cycle events and mood disorders, our hypothesis was that clinical features of BD, and particularly the occurrence of perinatal mood episodes, could be correlated with the timing of menarche.

The sample examined in our study was representative of the population of women with BD: consistently with previous literature, 58.3% of women included in the study had BD type II, whereas the median age of BD onset was 27 (12–72) (26, 27). Moreover, the mean age at menarche (12.6 ± 1.3 years) was in line with the mean age at menarche of general population (28–33). Spearman correlations showed that age at menarche was positively associated with the number of lifetime depressive episodes and the duration of untreated illness. These findings are worthy of interest, but this is the first study to show this relationship and further methodologically rigorous research is needed. The logistic regression showed that EM was associated with the greater odds of metabolic syndrome, as already observed in previous studies (34–38). The underlying pathophysiological mechanisms involved in the association between age at menarche and metabolic syndrome remain poorly understood: whether EM functions as a risk factor by itself or *via* sex hormones lifespan differences needs to be investigated. However, the most suggested explanation is that EM in adolescent and metabolic syndrome in adulthood is consequences of childhood obesity. Childhood adiposity might trigger puberty by adipocytes and related hormones (36). In addition, childhood obesity progresses to adult obesity and is subsequently related with metabolic syndrome (39). Although more studies are needed to deepen the underlying mechanisms, the association between age at menarche and metabolic disorders in women with BD should be taken into account when choosing the pharmacological treatments.

In order to examine the association between the age at menarche and the occurrence of peripartum mood episodes, the subgroup of women with children was analyzed (58.7% of the total sample). They do not present significant clinical differences compared to women without children except a lower rate of psychiatric comorbidities. About one-third (29.6%) of them had at least one mood episode with peripartum onset. The lack of data on the pharmacological treatments at the time of pregnancy makes it difficult to interpret this finding. However, the rate of peripartum episodes in our study is slightly higher than those found in samples of women taking medication during pregnancy and much lower than those of women who were medication free during pregnancy (40). In our sample, LM was associated with lower probability of mood episodes with peripartum onset. Given the lack of clinical predictors that may help to individualize the risk of perinatal recurrence in women with BD (41), this result is worthy of interest and deserves to be deepened in further studies.

The results should be considered in light of several limitations. First, the cross-sectional design of the study does not allow to infer causal relationship between studied variables. Although we can eliminate the risk of reverse bias for some characteristics such as age at menarche, the temporal order for other characteristics remains an unsolved problem. Moreover, there may be some unmeasured confounders such as medications we could not control for in our model: Variables such as age, duration of illness, and number of lifetime mood episodes could lead to different types of pharmacological treatments interfering with associations reported in the study. Finally, significant associations may have been lost for some variables due to the sample size. However, there are also few strengths: The missing data for studied variables was very low, models were adjusted for several confounders, and data allowed us to examine the associations with a large number of clinical characteristics.

## Conclusion

The results suggest that age at menarche may be associated with specific characteristics of women with BD: EM with higher probability of developing metabolic syndrome and LM with lower rate of peripartum mood episode. If confirmed by further studies, these findings might improve the ability to predict long-term BD outcomes and to choose tailored treatments for specific subgroups of patients.

## Data Availability Statement

The raw data supporting the conclusions of this article will be made available by the authors, without undue reservation.

## Ethics Statement

The studies involving human participants were reviewed and approved by Comitato Etico Interaziendale A.O.U. San Luigi Gonzaga di Orbassano AA.SS.LL. TO3-TO4-TO5. The patients/participants provided their written informed consent to participate in this study.

## Author Contributions

GR and GM designed the study. GR and EA collected the patients’ data. GR, EA, EM, and GDS managed literature search and analyzed the data. GR wrote the draft. All authors contributed to the article and approved the submitted version.

## Conflict of Interest

GR is/has been a speaker and/or consultant from Angelini, Janssen, Lundbeck, and Otsuka. GDS has been a speaker for Lundbeck. GM is/has been a consultant and/or a speaker and/or has received research grants from Angelini, Boheringer Ingelheim, FB-Health, Janssen, Lundbeck, Otsuka and, Innova Pharma.

The remaining authors declare that the research was conducted in the absence of any commercial or financial relationships that could be construed as a potential conflict of interest.

The handling editor declared a past co-authorship with one of the authors, GR.
